# Gallbladder Perforation: A 23-Year Study of 100 Patients and Evolving Management Strategies

**DOI:** 10.7759/cureus.108275

**Published:** 2026-05-04

**Authors:** Vivek Tandon, Deepak Govil, Supreet Kumar, Sonam Gupta, Suryalok Pratap Shah

**Affiliations:** 1 Surgical Gastroenterology, Medanta Hospital, Noida, IND

**Keywords:** biliary enteric fistula, complicated acute cholecystitis, complications of gallstone disease, delayed laparoscopic cholecystectomy, niemeier’s classification, percutaneous continuous drainage, perforation of gallbladder

## Abstract

Background: Gallbladder perforation (GBP) is an uncommon but clinically significant complication of gallstone disease, characterized by heterogeneous presentation, diagnostic ambiguity, and variable management strategies. Despite advances in imaging and surgical techniques, optimal treatment pathways remain ill-defined, particularly in distinguishing patients requiring urgent intervention from those suitable for conservative or delayed management.

Methods: A retrospective analysis was conducted of 100 patients diagnosed with GBP at a single tertiary care center over a 23-year period (2002-2025). Clinical presentation, comorbidity profile, imaging findings, perforation subtype, management strategies, and outcomes were systematically evaluated. Patients were classified according to the modified Niemeier classification. Temporal trends in diagnosis, intervention, and surgical approach were analyzed to assess evolving management patterns. Data were analyzed descriptively to identify clinically meaningful patterns.

Results: The cohort comprised 100 patients with a mean age of 61.1 years and a male predominance. Type II perforation was the most common subtype (62%), followed by Type III (34%) and combined Type II and III (4%), with no cases of Type I perforation observed. Preoperative diagnosis was achieved in 49% of cases, predominantly in Type II perforations, whereas Type III perforations were largely diagnosed intraoperatively. Imaging accuracy improved substantially in the later years of the study.

Percutaneous drainage was required in 11% of patients and was employed selectively in those with persistent sepsis or significant collections. The majority of patients were managed with initial conservative treatment followed by interval surgery. A progressive shift toward laparoscopic surgery was observed over time, with improved feasibility and outcomes in delayed settings. Type III perforations were associated with increased operative complexity and a higher requirement for additional procedures. Occult malignancy was identified in 2% of patients.

Conclusion: GBP represents a spectrum of diseases requiring individualized, physiology-driven management. Most Type II perforations can be managed conservatively with selective intervention, while delayed surgery facilitates safer and more effective minimally invasive treatment. Type III perforation, although technically demanding, can often be managed electively in stable patients. These findings support a tailored approach that prioritizes sepsis control, appropriate patient selection, and optimal timing of surgery over routine early escalation.

## Introduction

Gallbladder perforation (GBP) is an uncommon but potentially catastrophic complication of gallstone disease, first described by Duncan in the mid-19th century and later systematized by Niemeier into a clinico-pathological classification that continues to guide contemporary understanding [1,2]. Despite advances in diagnostic imaging, critical care, and minimally invasive surgery, GBP remains a diagnostic and therapeutic challenge, largely owing to its protean clinical presentation and frequent overlap with uncomplicated acute cholecystitis. Delayed recognition is associated with significant morbidity and mortality, particularly in elderly patients and those with multiple comorbidities [3].

The reported incidence of GBP among patients with acute cholecystitis varies between 1% and 11%, with wide heterogeneity across geographic regions and healthcare systems [3,4]. According to the modified Niemeier classification, perforations are categorized as free perforation into the peritoneal cavity (Type I), localized or contained perforation with pericholecystic abscess (Type II), and chronic perforation with cholecysto-enteric fistula formation (Type III) [2]. Among these, Type II perforation is consistently reported as the most common, whereas Type I perforation, though less frequent, carries the highest risk of sepsis and mortality [3,4].

Over the past decade, several large single-center series have advocated a “step-up” approach to management, emphasizing early sepsis control with antibiotics, percutaneous drainage (PCD), and biliary decompression, followed by delayed definitive surgery [4,5]. While this strategy has improved outcomes, important questions remain unanswered regarding the optimal selection of patients for percutaneous intervention, the necessity of emergency surgery in all cases, and the ideal timing and surgical approach for definitive cholecystectomy, particularly in Type II and Type III perforations. Moreover, most available studies are limited by relatively short study periods and do not adequately capture how evolving imaging modalities, surgical expertise, and perioperative care influence long-term management paradigms.

In this context, we present a 23-year single team experience of spontaneous GBP from a high-volume tertiary hepatopancreatobiliary (HPB) unit. The primary objectives of this study were to evaluate the incidence, clinical presentation, radiologic diagnosis, and management strategies of GBP over an extended period, with particular emphasis on Type II and Type III perforations. Secondary objectives included assessing temporal trends in surgical timing and approach, the role of selective PCD, and the incidence of occult malignancy in patients presenting with GBP. By analyzing outcomes across more than two decades, this study aims to elucidate evolving management paradigms and provide pragmatic, evidence-based insights into the contemporary treatment of GBP.

Given the retrospective design, the rarity of GBP, and the heterogeneity in clinical presentation and management over a 23-year period, this study was conceived as a descriptive, hypothesis-generating analysis aimed at characterizing institutional patterns and temporal trends rather than a hypothesis-testing comparative study.

## Materials and methods

Study design and patient selection

This retrospective observational study was conducted at a high-volume tertiary HPB center, Indraprastha Apollo Hospital, New Delhi. All consecutive patients diagnosed with spontaneous GBP between January 2002 and December 2025 were identified from a prospectively maintained institutional surgical database. Patients with GBP secondary to abdominal trauma, iatrogenic injury, or endoscopic interventions were excluded from the analysis. The study cohort comprised patients in whom the diagnosis of GBP was established either preoperatively on imaging or intraoperatively during surgical exploration. Clinical decision-making regarding imaging, PCD, and timing of surgery was individualized and based on institutional practice patterns rather than predefined protocolized thresholds.

Definitions and classification

GBP was classified according to the modified Niemeier classification into Type I (free perforation into the peritoneal cavity), Type II (contained perforation with localized pericholecystic collection or abscess), and Type III (chronic perforation with cholecysto-enteric fistula) [2]. Combined perforations demonstrating features of both Type II and Type III were recorded as a separate subgroup for analysis.

A preoperative diagnosis of GBP was defined as radiologic evidence of gallbladder wall discontinuity, pericholecystic abscess or collection, extraluminal gallstones, pneumobilia, or a fistulous communication identified on ultrasonography, computed tomography (CT), or magnetic resonance imaging (MRI).

Clinical evaluation and imaging

Baseline demographic characteristics, presenting symptoms, comorbid conditions, laboratory parameters, and imaging findings were retrieved from medical records. Ultrasonography was used as the initial imaging modality in all patients, followed by contrast-enhanced CT and/or MRI based on clinical assessment, imaging uncertainty, and suspicion of complications. Cross-sectional imaging (CT or MRI) was obtained in patients with persistent or worsening symptoms, inconclusive ultrasonography, suspicion of complications such as perforation or collection, or evidence of biliary obstruction. The diagnostic yield of imaging was assessed overall and stratified by type of perforation and time period to evaluate temporal trends and improvements in preoperative detection [6,7].

Management strategy

All patients were initially managed with broad-spectrum intravenous antibiotics and supportive care, with antibiotic selection guided by severity of infection and institutional antimicrobial protocols, and subsequently modified based on clinical response and microbiological data where available. PCD of pericholecystic or intra-abdominal collections was performed selectively in patients with persistent sepsis despite antibiotic therapy, large or loculated intra-abdominal collections on imaging, ongoing fever or leukocytosis, or poor physiological reserve precluding immediate surgical intervention. Routine cholecystostomy was not employed in this series.

Endoscopic retrograde cholangiopancreatography (ERCP) with biliary clearance or decompression was performed when indicated for associated choledocholithiasis or biliary obstruction. Definitive surgical management, including laparoscopic or open cholecystectomy with fistula dismantling and primary repair or closure of the involved viscus, with additional biliary or enteric procedures performed based on intraoperative findings was undertaken electively following resolution of the acute inflammatory process.

Interval surgery was defined as definitive operative management performed at least six weeks after the index presentation, after clinical stabilization, resolution of sepsis, optimization of comorbid conditions, and multidisciplinary perioperative assessment of surgical fitness.

Data collection and outcomes

Data collected included type of perforation, timing and modality of diagnosis, initial management approach, timing and type of definitive surgery, need for additional procedures, postoperative morbidity, length of hospital stay, and in-hospital mortality. Temporal trends in management strategies and outcomes were analyzed by comparing two eras (2002-2014 and 2015-2025) to reflect evolving diagnostic capabilities and surgical practice. Data completeness was ensured through cross-verification with institutional records. Missing data for individual variables were handled by case-wise exclusion without imputation. Patients lost to follow-up after initial management were analyzed separately and excluded from definitive surgical outcome analysis. The study was not powered for comparative statistical analysis between subgroups. Outcomes assessed were limited to in-hospital morbidity and mortality. Long-term follow-up and standardized morbidity grading systems were not uniformly available and are acknowledged as a limitation.

Statistical analysis

Continuous variables are expressed as mean with range or median with range, as appropriate. Categorical variables are presented as frequencies and percentages. Given the retrospective design, the relatively small sample size for subgroup comparisons, and the substantial temporal heterogeneity in imaging modalities, perioperative care, and surgical practices over the 23-year study period, analyses were primarily descriptive and exploratory. Formal hypothesis testing, regression modeling, and adjustment for temporal confounders were not performed, as such analyses without appropriate control for these variables could yield misleading inferences. The findings are therefore presented as observational trends intended to generate clinically relevant hypotheses for future prospective or multicenter studies. Statistical summaries were generated using IBM SPSS Statistics for Windows, Version 25 (Released 2017; IBM Corp., Armonk, New York, United States).

## Results

Patient cohort and incidence

Between January 2002 and December 2025, approximately 4,500 cholecystectomies were performed at our institution. During this period, 100 patients were diagnosed with spontaneous GBP, corresponding to an overall incidence of ~2.2% among surgically managed gallbladder disease. The cohort comprised 63 male patients (63%) and 37 female patients (37%), with a mean age of 61.10 years (range: 20-92 years). Gallstones were present in all patients (100%), while associated common bile duct (CBD) stones were identified in 23 patients (23.0%).

Clinical presentation and comorbidity profile

Abdominal pain was the universal presenting symptom (100%). Fever was present in 53 patients (53.0%), and 26 patients (26.0%) presented with jaundice. Among jaundiced patients, 15 of 26 (57.7%) had associated CBD stones. No comorbidities were present in 38 patients (38.0%). A single comorbidity was present in 23 patients (23.0%), two comorbidities in 19 patients (19.0%), and three or more comorbidities in 20 patients (20.0%). Patients with Type III perforation demonstrated a higher overall comorbidity burden compared with those with Type II perforation, whereas patients without comorbidities were more frequently represented in the Type II subgroup. The baseline demographic and clinical characteristics of the study population are summarized in Table 1.

**Table 1:** Baseline Demographic and Clinical Characteristics of Patients with Gallbladder Perforation (n = 100) CBD: Common bile duct

Variable	Value
Total patients	100
Mean age (years), range	61.10 (20–92)
Sex (Male: Female)	63: 37
Gallstones	100 (100%)
CBD stones	23 (23.0%)
Abdominal pain	100 (100%)
Fever	53 (53.0%)
Jaundice	26 (26.0%)
No comorbidity	38 (38.0%)
One comorbidity	23 (23.0%)
Two comorbidities	19 (19.0%)
Three or more comorbidities	20 (20.0%)

Type and distribution of GBP

According to the modified Niemeier classification, no patient in this 23-year cohort had Type I (free) GBP. The distribution of GBP types according to the modified Niemeier classification is presented in Table 2. The distribution of cholecysto-enteric fistula types among patients with Type III perforation is detailed in Table 3.

**Table 2:** Type and Distribution of Gallbladder Perforation According to Modified Niemeier Classification (n = 100)

Perforation Type	Number (%)
Type I	0 (0%)
Type II (contained perforation)	62 (62.0%)
Type III (cholecysto-enteric fistula)	34 (34.0%)
Type II + III (Combined)	4 (4.0%)
Total	100 (100%)

**Table 3:** Distribution of Cholecysto-enteric Fistula Types in Patients with Type III Gallbladder Perforation (n = 38)

Fistula Type	Number (%)
Cholecystoduodenal	33 (86.8%)
Cholecystocolic	3 (7.9%)
Cholecystogastric	2 (5.3%)

A significant temporal evolution in perforation patterns was observed. Temporal trends in the distribution of GBP types over the study period are shown in Table 4.

**Table 4:** Temporal Trends in Gallbladder Perforation Types Over the Study Period (n = 100)

Period	Type II	Type III	Type II + III	Total
2002–2014	18	23	0	41
2015–2025	44	11	4	59

This shift reflects an increase in Type II perforations and a decline in Type III perforations in the later era, coinciding with improved imaging availability and lower threshold to order them leading to earlier diagnosis of contained perforations.

Preoperative diagnosis and imaging performance

A preoperative radiologic diagnosis of GBP was achieved in 49 of 100 patients (49%). The distribution of imaging modalities contributing to preoperative diagnosis is summarized in Table 5.

**Table 5:** Preoperative Diagnostic Modalities in Gallbladder Perforation (n = 100)

Imaging Modality	Number
Ultrasonography alone	2
CT alone	32
MRI alone	5
CT + MRI	10
Total diagnosed	49

One patient undergoing CT for suspected small bowel obstruction demonstrated pneumobilia raising suspicion of cholecysto-enteric fistula, which was confirmed intraoperatively as Type III (cholecysto-duodenal).

Preoperative diagnosis was achieved predominantly in Type II perforations. In contrast, Type III perforations were overwhelmingly diagnosed intraoperatively, with only one case suspected preoperatively due to pneumobilia detected on CT performed for small bowel obstruction. The temporal improvement in preoperative diagnostic yield is demonstrated in Table 6.

**Table 6:** Temporal Improvement in Preoperative Diagnosis

Period	Diagnosed
2002–2013	5
2014–2025	44

In 2002-2013, there were five preoperative diagnoses (CT 3, CT+MRI 1, USG 1), and in 2014-2025, there were 44 preoperative diagnoses (CT 29, CT+MRI 9, MRI 5, USG 1).

Initial management and role of PCD

All patients received initial conservative management with antibiotics and supportive care. PCD was required in 11 patients (11.0%). Outcomes following PCD are detailed in Table 7.

**Table 7:** Outcomes Following Percutaneous Drainage (n = 11)

Outcome	Number
Proceeded to surgery	3
Mortality	1
Lost to follow-up	7

Definitive surgical management

The overall management outcomes in the study cohort are summarized in Table 8.

**Table 8:** Overall Management Outcomes in the Study Cohort PCD: Percutaneous drainage

Outcome	Number
Open surgery	37
Laparoscopic surgery	48
No procedure	7
Lost to follow-up (after PCD)	7
Mortality (after PCD)	1
Total	100

Seven patients did not undergo definitive surgery after initial conservative treatment: one had GBP in the setting of road traffic accident with extensive associated injuries; four were deemed unfit due to comorbidities; and two were lost to follow-up for unclear reasons.

Evolution of the surgical approach over time

A progressive shift toward minimally invasive surgery was observed. The evolution of surgical approach over time is illustrated in Table 9. In the last 26 patients (post-2019), a policy of longer wait from index episode was adopted (mean 40 days). Management in this subgroup included laparoscopic surgery in 15 patients (Type II: 14; Type III: 1), open surgery in seven patients (Type II: 4; Type III: 3), no definitive procedure in three patients and PCD-only management in one patient.

**Table 9:** Management Outcomes Stratified by Type of Gallbladder Perforation (n = 100)

Period	Open	Laparoscopic	No procedure	Lost to follow-up	Mortality	Total
2002–2014	20	17	3	1	0	41
2015–2025	17	31	4	6	1	59

Management according to the perforation type

Management outcomes stratified by the type of GBP are presented in Table 10.

**Table 10:** Type of Procedure as Per Perforation Type PCD: Percutaneous drainage

Procedure/Outcome	Type II (n=62)	Type III (n=34)	Type II+III (n=4)	Total
Open surgery	13	22	2	37
Laparoscopic surgery	34	12	2	48
PCD	7	0	0	7
No procedure	7	0	0	7
Mortality	1	0	0	1
Total	62	34	4	100

Additional procedures and complexity indicators

Additional procedures were more frequent in Type III perforation. Additional procedures by perforation type are presented in Table 11. 

**Table 11:** Additional Procedures Performed According to Type of Gallbladder Perforation (n = 100) Values are presented as the number of procedures. Type II: contained perforation; Type III: cholecysto-enteric fistula; Type II + III: combined perforation. CBD: common bile duct; CDD: choledochoduodenostomy; HJ: hepaticojejunostomy; GJ: gastrojejunostomy.

Additional Procedure	Type II (n = 62)	Type III (n = 34)	Type II + III (n = 4)	Total
CBD exploration	1	1	1	3
CBD repair	0	1	0	1
CDD	1	2	0	3
HJ	0	2	0	2
GJ	0	2	0	2
Feeding jejunostomy	4	15	1	20
Liver wedge	3	3	0	6

Incidence of underlying malignancy

Unexpected malignancy was identified on final histopathology in two patients (2.0%), one with Type II and one with combined Type II + III perforation. Both had undergone PCD followed by laparoscopic surgery.

## Discussion

GBP continues to represent a complex clinical entity, not because of its frequency, but due to its heterogeneous presentation, variable natural history, and persistent lack of consensus regarding optimal management. The present 23-year single-team experience involving 100 patients offers a robust longitudinal perspective that permits not only description of outcomes, but also critical appraisal of evolving diagnostic and therapeutic strategies in comparison with contemporary literature.

Absence of Type I GBP: a distinctive phenotypic pattern

One of the most striking and consistent observations in this expanded cohort is the complete absence of Type I (free) GBP over a 23-year period. This finding contrasts sharply with contemporary series, where Type I perforation accounts for 8-15% of cases and is associated with the highest morbidity and mortality [3-5]. In the step-up cohort reported by Gupta et al., Type I perforation constituted 8.6% of patients and was strongly linked to diabetes, hypertension, acute kidney injury, and mortality [4].

In the context of our cohort, the absence of Type I perforation is best interpreted as a function of referral dynamics and disease biology rather than as a true epidemiologic rarity. Patients with free perforation may present directly to emergency departments, undergo urgent surgery at peripheral centers, or succumb prior to referral. Similar under-representation of Type I perforation has been described in other tertiary referral-based series, underscoring how institutional case-mix and pre-hospital pathways shape the observed perforation spectrum [8,9].

Type II perforation: predominantly managed with conservative and selective interventions

Type II perforation remains the dominant phenotype in our series, accounting for 62% of cases, consistent with existing literature [3,4,10]. Importantly, the enlarged dataset further reinforces the distinctive management philosophy adopted in our unit. While Gupta et al. utilized PCD in nearly 28% of Type II cases [4], and other authors advocate liberal image-guided drainage within step-up protocols [11,12], only 11% of patients in our series required PCD, and no patient underwent routine cholecystostomy.

The updated results suggest that, in our cohort, a substantial proportion of Type II perforations were initially managed with antibiotics and supportive care, with PCD reserved for a minority of patients with persistent sepsis or large, loculated collections. This selective approach reduces procedure-related morbidity, avoids prolonged catheter dependence, and mitigates loss to follow-up, an under-reported but clinically relevant issue observed in our PCD subgroup. This observed pattern reflects an institutional, physiology-guided approach to management rather than a protocolized strategy and should be interpreted as a descriptive trend rather than evidence of superiority over alternative management pathways. 

Diagnostic yield of imaging and the persistent blind spot of type III perforation

The overall preoperative diagnostic rate of GBP in our series (49%) aligns with previously reported figures ranging from 36% to 60% [3,4,13]. Crucially, the diagnostic pattern remained unchanged: preoperative diagnosis was achieved predominantly in Type II perforations, while Type III perforations were overwhelmingly identified intraoperatively. Only a single Type III case was suspected preoperatively, prompted by pneumobilia on CT performed for suspected small bowel obstruction.

This observation closely mirrors the experience of Gupta et al., where approximately 90% of Type III perforations were diagnosed only at surgery [4]. Despite widespread availability of high-resolution CT and MRI in the later years of the study, cross-sectional imaging continued to demonstrate limited sensitivity for detecting cholecysto-enteric fistulas [13,14]. These findings reinforce the notion that Type III perforation remains largely a surgical diagnosis, necessitating a high index of suspicion when dense adhesions, pneumobilia, or distorted anatomy are encountered intraoperatively.

Timing of surgery: interval management as an enabler of minimally invasive surgery

A defining feature of the present series is the intentional use of interval surgery, with definitive intervention typically performed 5-8 weeks after index presentation, with a mean interval of approximately 40 days. Earlier series favored early or emergency surgery due to concerns regarding ongoing sepsis and technical difficulty [10,15]. In contrast, our observations suggest that delayed surgery in our cohort was associated with progressively increasing laparoscopic success, with a marked shift toward minimally invasive surgery in the later era and a 100% laparoscopic completion rate in the last 20 cases.

Gupta et al. reported low laparoscopic completion rates in Type II perforation and universal open surgery in Type III disease [4], while other series have documented high conversion rates and frequent subtotal cholecystectomy in acute settings [5,15]. The present observations suggest that delayed surgery in our cohort was associated with resolution of inflammation, clearer tissue planes, and safer dissection, challenging the assumption that early surgery is universally beneficial in GBP. These findings represent descriptive associations within this cohort and do not establish a causal advantage of delayed over early intervention.

Type III perforation: from surgical emergency to elective complexity

Historically, Type III perforation has been regarded as a surgical emergency, often associated with gallstone ileus or uncontrolled sepsis [16]. In the present cohort, only one patient required emergency surgery for gallstone ileus, while the majority of Type III perforations were managed electively. This pattern diverges from older literature but aligns with more recent reports suggesting that many cholecysto-enteric fistulas evolve gradually and may be addressed in a controlled setting in selected clinically stable patients [17,18].

The higher incidence of additional procedures, particularly feeding jejunostomy and biliary or enteric reconstruction in Type III perforation, underscores the inherent technical complexity of these cases. The observed reduction in adjunctive procedures over time likely reflects improved preoperative optimization, maturation of surgical judgment, and increasing familiarity with advanced laparoscopic techniques.

GBP and occult malignancy

In the final cohort, occult malignancy was identified in 2.0% of patients, both detected following interval surgery after initial PCD. This finding remains consistent with earlier observations that gallbladder malignancy may masquerade as perforation, particularly in Type II disease [4,19]. The updated data further reinforce the oncologic risk of indiscriminate emergency surgery and support a staged approach, when clinically feasible, to preserve oncologic principles and permit appropriate surgical planning.

This study has several important limitations. Its retrospective design introduces inherent selection and information bias. As a tertiary referral-center experience, the cohort is subject to referral bias, which may partly explain the absence of Type I perforation. The 23-year study period introduces significant temporal confounding, as advances in imaging modalities, perioperative care, anesthesia, and surgical expertise evolved substantially over time. Management strategies were not protocolized by predefined clinical or biochemical thresholds, and decisions regarding imaging, PCD, and timing of surgery were individualized based on institutional practice patterns, introducing potential selection bias and limiting generalizability. Formal hypothesis testing, regression modeling, and adjustment for temporal confounders were not performed; therefore, observed patterns should be interpreted as descriptive trends rather than evidence of statistically validated superiority. Long-term outcomes and standardized morbidity grading were not uniformly available. These limitations should be considered when interpreting the findings of this study.

## Conclusions

GBP represents a heterogeneous clinical entity whose management must be individualized rather than protocolized. This 23-year single-center experience demonstrates that the contemporary phenotype of GBP in a tertiary referral setting is dominated by contained (Type II) and chronic fistulous (Type III) perforations, with free perforation (Type I) being conspicuously absent. This observation underscores the influence of referral patterns, disease biology, and early containment on the clinical spectrum of GBP encountered in specialized centers. The findings of this study challenge the prevailing notion that GBP uniformly mandates urgent surgical intervention. Most Type II perforations can be managed successfully initially with antibiotics and supportive care alone, with selective use of percutaneous drainage reserved for patients with persistent sepsis or large collections. Routine cholecystostomy is rarely required. When definitive surgery is indicated, a deliberate interval strategy facilitates resolution of inflammation and enables safe minimally invasive surgery, as reflected by progressively increasing laparoscopic completion rates over time.

Type III perforation, while surgically complex, is not invariably an emergency. In the absence of gallstone ileus or uncontrolled sepsis, planned surgery is feasible. Advances in imaging, perioperative care, and surgical expertise have reduced the need for extensive adjunctive procedures and have transformed Type III perforation from an emergent catastrophe into a manageable elective challenge in selected patients. The identification of occult gallbladder malignancy in a subset of patients further reinforces the importance of a staged, physiology-driven approach that preserves oncologic principles and avoids indiscriminate emergency surgery. Collectively, these findings advocate for a tailored management paradigm in GBP, one that prioritizes sepsis control, judicious intervention, optimal timing of surgery, and surgical judgment over reflex escalation.
